# In Vitro Studies on Antioxidant and Anti-Parasitic Activities of Compounds Isolated from *Rauvolfia caffra* Sond

**DOI:** 10.3390/molecules25173781

**Published:** 2020-08-20

**Authors:** Dorcas B. Tlhapi, Isaiah D. I. Ramaite, Chinedu P. Anokwuru, Teunis van Ree, Heinrich C. Hoppe

**Affiliations:** 1 Department of Chemistry, University of Venda, Private Bag X5050, Thohoyandou 0950, South Africa; dorcastlhapi@gmail.com (D.B.T.); anokwuruchi@gmail.com (C.P.A.); Teuns.VanRee@univen.ac.za (T.v.R.); 2 Department of Biochemistry and Microbiology, Rhodes University, Grahamstown 6140, South Africa; H.Hoppe@ru.ac.za

**Keywords:** bioactive compounds, *Rauvolfia caffra* Sond, antioxidant activity, antitrypanosomal activity

## Abstract

As part of an ongoing study of natural products from local medicinal plants, the methanol extract of stem bark of *Rauvolfia caffra* Sond was investigated for biological activity. Column chromatography and preparative thin-layer chromatography were used to isolate lupeol (**1**), raucaffricine (**2**), *N*-methylsarpagine (**3**), and spegatrine (**4**). The crude extract, fractions and isolated compounds were tested for anti-oxidant, antitrypanosomal and anti-proliferation activities. Two fractions displayed high DPPH (2,2-diphenyl-1-picrylhydrazyl) free radical scavenging activity and reducing power with IC_50_ (The half maximal inhibitory concentration) and IC_0.5_ values of 0.022 ± 0.003 mg/mL and 0.036 ± 0.007 mg/mL, and 0.518 ± 0.044 mg/mL and 1.076 ± 0.136 mg/mL, respectively. Spegatrine (**4**) was identified as the main antioxidant compound in *R. caffra* with IC_50_ and IC_0.5_ values of 0.119 ± 0.067 mg/mL and 0.712 ± 0 mg/mL, respectively. One fraction displayed high antitrypanosomal activity with an IC_50_ value of 18.50 μg/mL. However, the major constituent of this fraction, raucaffricine (**2**), was not active. The crude extract, fractions and pure compounds did not display any cytotoxic effect at a concentration of 50 μg/mL against HeLa cells. This study shows directions for further in vitro studies on the antioxidant and antitrypanosomal activities of *Rauvolfia caffra* Sond.

## 1. Introduction

Trypanosomiasis is a fatal disease caused by the genus *Trypanosoma*, affecting humans and animals in many African countries [1,2,3]. The protozoan parasites are transmitted by the bite of infected flies of the Tabanidae family [4,5]. The World Health Organization (WHO) estimates that over 70 million people in Sub-Saharan Africa are exposed to the risk of contracting this disease, which is deadly if left untreated [6]. Treatment of this condition has been a challenge [7], although the recent introduction of fexinidazole promises to alleviate some of the efficacy, toxicity and administration problems experienced with historical drug treatments [8]. Nonetheless, the propensity of pathogens to generate resistance motivates the ongoing search for novel therapeutic drugs to combat this dreadful pathogen. Natural products from medicinal plants have played a vital role in the treatment of trypanosomiasis in various African countries [2]. Therefore, medicinal plants have a great potential to provide new antiprotozoal treatments.

Oxidative stress is an imbalance between free radicals and antioxidants in the body, causing DNA damage, protein oxidation, lipid peroxidation, regulation of intracellular signal transduction and physiological adaptation phenomena [9,10]. Free radicals are unstable molecules that initiate mitochondrial oxidative phosphorylation, prostaglandin synthesis and phagocytosis [11]. The human body is always at risk of being exposed to external sources of free radicals that could affect the functioning of the cells, but the body has developed a natural antioxidant defence system to prevent oxidative stress damage [10]. Traditional medicinal plants have always been considered as a source of natural antioxidants [12], which are highly effective in preventing the processes of oxidation by neutralizing free radicals [13]. Numerous medicinal plants are used as alternative drugs and sources of novel plant-derived constituents that could be leads for treatments against numerous ailments [14]. However, many compounds extracted from traditional plants have not been thoroughly studied for toxicity.

In our continuing search for biologically active metabolites of traditional medicinal plants from the Venda area in South Africa, we investigated *Rauvolfia caffra*, which is widely used in medicine in Sub-Sahara African communities to treat a range of ailments such as toothaches, hysteria, epilepsy, eye diseases, skin wounds, urticaria, severe abdominal pains, diarrhoea, headaches, constipation, irregular periods, swollen legs, palpitation of the heart, insomnia, insecurity, restlessness, anxiety and earache [15,16,17,18,19,20]. Previously, we isolated three indole alkaloids (raucaffricine, *N*-methylsarpagine and spegatrine) and one terpenoid (lupeol) from the stem bark of *Rauvolfia caffra* and found that the major constituents do not contribute to the antiplasmodial activity of *R. caffra* [21].

The main constituents isolated and identified from the stem bark of *R. caffra* are macrocaffrine, yohimbine, sarpagan, akuammicine, dihydroindole, indolenine, peraksine, oxindole, anhydronium, ajmaline, ajmalicine and geissoschizol. These alkaloids also have pharmacological activities, such as antimicrobial, anti-inflammatory, antimalarial, antioxidant, antitumor and antidiabetic properties [22,23]. Although herbal practitioners use *Rauvolfia caffra* in South Africa and some African countries for the treatment of a wide range of diseases, there are a limited number of studies into the chemistry of the underground and aerial parts of *R. caffra* as well as the bioactivity of the extracts. Therefore, we decided to isolate compounds from the stem bark, identify the major chemical constituents present and determine if they are responsible for the bioactivity of the plant. Our results confirm the reported antioxidant activity of the crude methanol extracts from *Rauvolfia caffra* [22,23]. However, isolated compounds from this plant have not yet been studied for their antioxidant activity. Moreover, the antitrypanosomal activity of the crude methanol extracts and isolated bioactive compounds from *Rauvolfia caffra* have never been evaluated. Therefore, this study is the first survey to detect significant in vitro antioxidant and antitrypanosomal activities of *R. caffra*.

## 2. Results and Discussion

### 2.1. Antioxidant Activity

In the DPPH (2,2-diphenyl-1-picrylhydrazyl) free radical scavenging activity test, fraction F_4_ exhibited the highest free radical scavenging activity with an IC_50_ (50% inhibitory concentration) value of 0.022 ± 0.003 mg/mL while Fraction F_3_ displayed the lowest free radical scavenging activity with an IC_50_ value of 1.143 ± 0.478 mg/mL, as shown in Table 1. In the ferric reducing power assay, fraction F_3_ displayed the lowest reducing power activity with an IC_0.5_ value of 2.151 ± 0.372 mg/mL while fraction F_4_ exhibited the highest reducing power activity with an IC_0.5_ value of 0.518 ± 0.044 mg/mL. Therefore, fraction F_4_ exhibited the highest antioxidant activity in the DPPH free radical scavenging assay and reducing power assay. According to a literature survey, the biological activities of raucaffricine and *N*-methylsarpagine, which were isolated from fractions F_3_ and F_4_, respectively, have never been evaluated. In our study the antioxidant activities of raucaffricine and *N*-methylsarpagine could not be tested due to insufficient amounts of material after chemical analysis. However, spegatrine (**4**), the main constituent of fraction F_5_, exhibited antioxidant activity; this is the first time that this activity is being reported for spegatrine. Lupeol (**1**), the main constituent of fraction F_1_, is a known anti-oxidant [24], explaining the antioxidant activity of fraction F_1_.

### 2.2. Antitrypanosomal Activity

**Figure 1:**
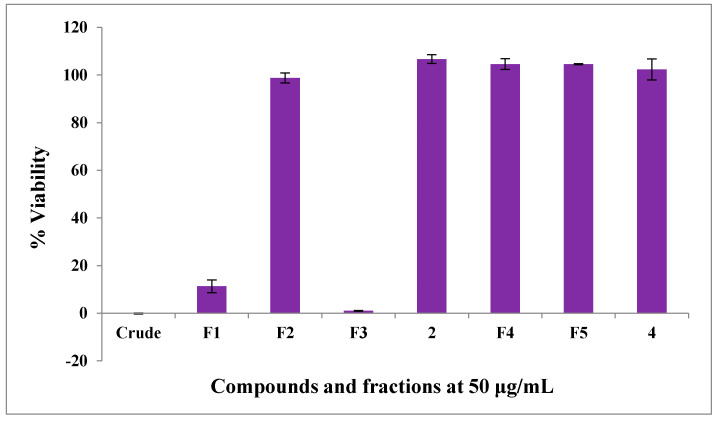
Antiparasitic activity against *T. brucei*: Crude—crude extract; F1—Fraction F_1_; F2—Fraction F_2_; F3—Fraction F_3_; F4—Fraction F_4_; F5—Fraction F_5_; 2—raucaffricine (**2**) and 4—spegatrine (**4**) expressed as % parasite viability ± standard deviation.

The antitrypanosomal activity of the crude extract, fractions F_1_, F_2_, F_3_, F_4_, F_5_, raucaffricine (**2**) and spegatrine (**4**) was determined against *Trypanosoma brucei brucei* (427 strain) parasites in in vitro cultures. The crude extract, fractions F_1_ and F_3_ strongly affected the viability of the trypanosomes at the tested concentration (50 μg/mL), with viabilities of −0.1 ± 0.2%, 11.3 ± 2.7% and 1.0 ± 0.1%, respectively, as shown in Figure 1. In contrast, at the same concentration, raucaffricine (**2**), isolated from F_3_ did not decrease the viability of the trypanosomes (106.7 ± 1.8%, Figure 1), showing that the major compound of this fraction did not contribute to the antitrypanosomal activity of the plant. To our knowledge this is the first report of significant antitrypanosomal activity of *R. caffra*.

**Figure 2:**
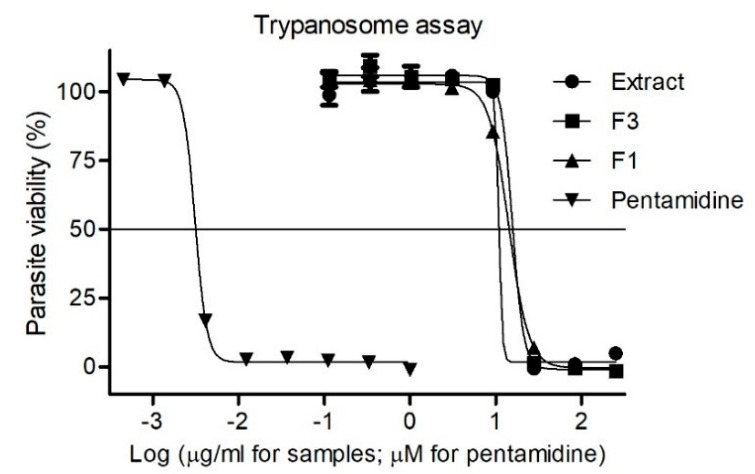
Dose-response curves for the trypanosome assay: Extract—crude extract; F3—Fraction. F_3_ and F1—Fraction F_1_ expressed as % parasite viability ± standard deviation.

As shown in Figure 2, the crude extract, fractions F_1_ and F_3_ displayed the highest antiparasitic activity with IC_50_ values of 14.2 μg/mL, 18.5 μg/mL, and 15.6 μg/mL, respectively. The pentamidine used as reference had an IC_50_ value of 0.003 μM. The antiprotozoal potential of lupeol (**1**), the main compound from fraction F_1_, has been reported before [25].

### 2.3. Anti-Proliferation Activity

**Figure 3:**
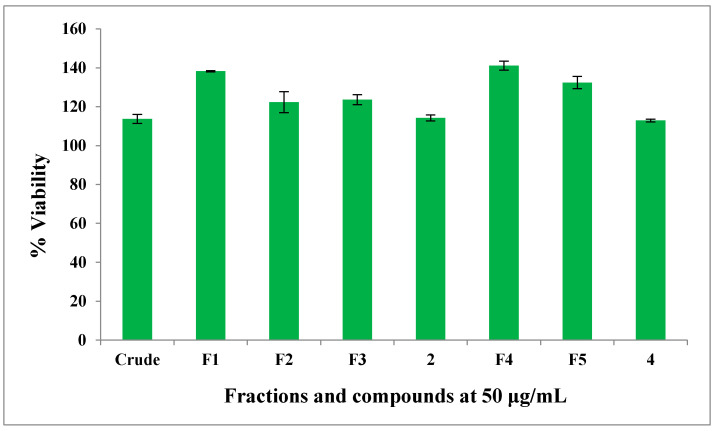
Anti-proliferation activity against HeLa cells: Crude—crude extract; F1—Fraction F_1_; F2—Fraction F_2_; F3—Fraction F_3_; F4—Fraction F_4_; F5—Fraction F_5_; 2—raucaffricine (**2**) and 4—spegatrine (**4**) expressed as % HeLa cell viability ± standard deviation.

The cell toxicity assay (CTA) was used to determine the cytotoxic abilities of the crude extract, fractions F_1_, F_2_, F_3_, F_4_ and F_5_, raucaffricine (**2**) and spegatrine (**4**) against HeLa (human cervix adenocarcinoma) cells. Briefly, HeLa cells were cultured with the respective samples for 48 h and the remaining percentage cell viability relative to untreated control cells determined using resazurin. The standard drug emetine displayed an IC_50_ value of 0.026 μM, whereas all the tested samples did not cause any cytotoxic effects at a concentration of 50 μg/mL, as shown in Figure 3. A previous study showed that *R. caffra* has anti-proliferation activity. The MeOH/CH_2_Cl_2_ stem bark extract exhibited activity against the proliferation of Vero cells. However, the MeOH/CH_2_Cl_2_ stem bark extract did not show significant activity against proliferation of RD and Hep-G2 cells [26]. The anti-proliferation activity of *R. caffra* may be due to its active constituents, and these effects may be further influenced by the type of extraction and solvent used.

In the literature, the anti-proliferation activity of isolated compounds from *Rauvolfia caffra* has not been determined using HeLa cells. Therefore, this study is the first survey that assesses the important anti-proliferation activity of *R. caffra* against these human cells.

## 3. Materials and Methods

### 3.1. General Experimental Procedure

All chemicals used were analytical grade purchased from Sigma-Aldrich (Darmstadt, Germany). Silica gel (0.063–0.2 mm) and Sephadex LH-20 (Sigma-Aldrich, Darmstadt, Germany) were used as stationary phases and solvent mixtures described below were used as mobile phase in the chromatographic separations. Thin layer chromatography plates packed with silica gel (normal or reversed phase), were used to locate major components of the fractions.

#### 3.1.1. High-Resolution Mass Spectrometry

A Waters Synapt G2 Quadrupole time-of-flight (QTOF) mass spectrometer (MS) (Thermo Fisher Scientific, Waltham, MA, USA) connected to a Waters Acquity ultra-performance liquid chromatograph (UPLC) (Waters, Milford, MA, USA) was used for direct injection high-resolution mass spectrometric analysis. One µL of sample was injected into a stream of 60% acetonitrile and 40% dilute (0.1%) aqueous formic acid. This conveyed the sample directly to the QTOF mass spectrometer where data were acquired using both positive and negative electrospray ionisation. The following MS settings were used: cone voltage of 15 V, desolvation temperature of 275 °C, desolvation gas at 650 L/h, and the rest of the MS settings optimized for best resolution and sensitivity.

#### 3.1.2. Infrared Spectroscopy

Attenuated total reflection (ATR) infrared (IR) spectra were recorded on an Alpha Fourier transform infrared (FTIR) spectrometer (Bruker, Fällanden, Switzerland).

#### 3.1.3. Nuclear Magnetic Resonance (NMR) Spectroscopy

^1^H- and ^13^C-nuclear magnetic resonance (NMR) spectra were recorded at 400 MHz and 100 MHz, respectively, with an Avance 400 spectrometer (Bruker, Fällanden, Switzerland) using residual undeuterated solvent as the internal standard.

### 3.2. Plant Collection and Preparation

The stem bark of *R. caffra* was collected at the University of Venda Campus, located at 22°58′32″ South and 30°26′40″ East in Thohoyandou, Limpopo Province, South Africa in January 2016. Botanical identification was provided by Prof. Tshisikhawe, a botanist in the Department of Botany at the University of Venda, and a voucher specimen (BD 001) was deposited. The plant samples were air-dried for two months and the dry samples were ground to fine powder using an industrial blender (NETZSCH, Selb, Germany).

### 3.3. Extraction of Plant Material

About 1.7 kg ground stems of *Rauvolfia caffra* were soaked with 2 L methanol for 48 h at room temperature. The methanol extract was filtered, and then concentrated using a rotary evaporator (BÜCHI Labortechnik AG, Flawil, Switzerland) at 50 °C to obtain 49.2 g of dried extract. The crude methanol extract (49.2 g) was subjected to column chromatography over silica gel [27].

The extract was eluted initially with hexane and the solvent polarity was increased gradually with ethyl acetate and finally methanol, yielding 17 fractions. Fractions with similar TLC (Thin-layer chromatography) profiles, i.e., containing the same compounds but in varying concentrations, were combined and concentrated to dryness on a rotary evaporator giving a total of 8 fractions coded as F_A_-F_H_. F_A_ was obtained with hexane/ethyl acetate (70:30); F_B_ and F_C_ were obtained with hexane/ethyl acetate (30:70); F_D_ was obtained with ethyl acetate (100%); F_E_ and F_F_ were obtained with ethyl acetate/methanol (70:30); and F_G_ and F_H_ were obtained with ethyl acetate/methanol (30:70). The collected fractions were monitored on TLC plates.

### 3.4. Isolation and Purification of Compounds

Fractions F_B_, F_E_, F_F_ and F_G_ were further fractionated using column chromatography since they contained a large amount of material compared to fractions F_A_, F_C_, F_D_ and F_H_.

Fraction F_B_ (1.42 g) was subjected to Sephadex LH-20 column chromatography; the column was eluted with CH_2_Cl_2_/MeOH (50:50) followed by an increasing gradient of CH_2_Cl_2_/MeOH (up to 10:90) to obtain F_1_ (1 g). Fraction F_E_ (4 g) was subjected to silica gel column chromatography; the column was eluted using CH_2_Cl_2_/MeOH (50:50) followed by an increasing gradient of CH_2_Cl_2_/MeOH (up to 10:90) to obtain 2 subfractions, F_2_ (1.51 g) and F_3_ (1.55 g). Fraction F_F_ (4 g) was also subjected to silica gel column chromatography and the column was eluted using *n*-C_6_H_12_/EtOAc (50:50) followed by an increasing gradient of *n*-C_6_H_12_/EtOAc (up to 30:70) to obtain 1 subfraction, F_4_ (3 g). Fraction F_G_ (5.6 g) was subjected to Sephadex LH-20 column chromatography; the column was eluted using CH_2_Cl_2_/MeOH (50:50) followed by an increasing gradient of CH_2_Cl_2_/MeOH (up to 10:90) to obtain F_5_ (5 g). The collected fractions were monitored on TLC plates. The thin layer chromatograms were developed in a solvent system of ethyl acetate/methanol/water (EMW 81:11:8). A natural product staining solution (1 g methanolic diphenylboric acid, 100 mL methanol, 5 mL PEG 400 and 95 mL ethanol) was used to visualize compounds on a TLC plate.

**Figure 4:**
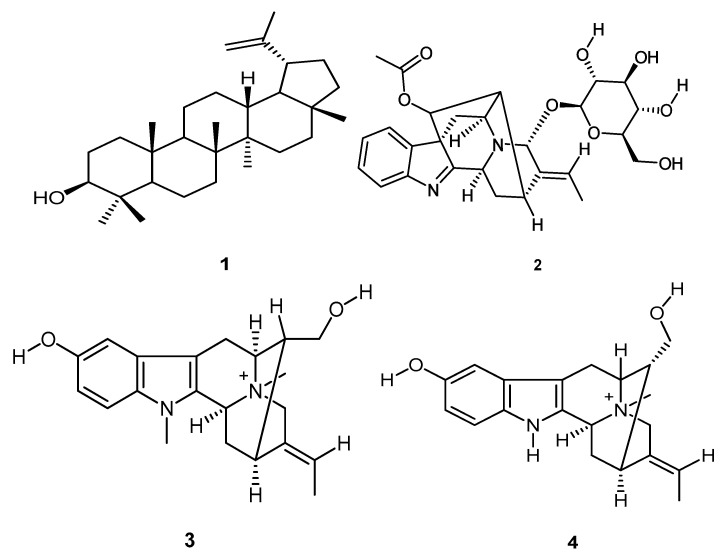
Compounds isolated from *R. caffra* extract: lupeol (**1**), raucaffricine (**2**), *N*-methylsarpagine (**3**) and spegatrine (**4**).

Fraction F_1_ (0.5 g) was subjected to preparative TLC (normal phase) to obtain compound **1** (0.230 g, Figure 4). Fraction F_2_ (0.5 g) was also subjected to preparative TLC (reversed phase) to obtain fraction F_2a_ (0.2102 g), an intractable mixture. Fraction F_3_ (0.5 g) was further purified using semi-preparative HPLC to yield compound **2** (0.019 g). Fraction F_4_ (0.5 g) was also subjected to preparative TLC (reversed phase) to obtain compound **3** (0.1238 g). F_5_ (1 g) was further purified using semi-preparative HPLC to obtain compound **4** (0.086 g). Compounds **1** [25], **2** [28], **3** [27], and **4** [27] are known compounds.

#### 3.4.1. Lupeol (**1**)

IR: 3000 cm^−1^ (O-H). ^1^H-NMR (400 MHz, CD_3_OD): δ_H_ 4.7 (1H, s, 29a-H), 4.6 (1H, s, 29b-H), 3.18 (1H, m, 3-H), 2.38 (1H, m, 19-H), 1.91 (1H, m, 21-H), 1.70 (3H, s, 30-H), 1.60 (1H, m, 2-H), 1.39 (1H, m, 18-H), 1.30 (1H, m, 9-H), 1.04 (3H, s, _C_ 26-H), 1.0 (3H, s, 23-H), 0.97 (3H, s, 27-H), 0.86 (3H, s, 25-H), 0.77 (3H, s, 24-H) ppm. ^13^C-NMR (100 MHz, CD_3_OD): δ_C_ 150.95 (C-20), 108.54 (C-29), 78.27 (C-3), 55.48 (C-5), 50.64 (C-9), 49.16 (C-18), 42.17 (C-14), 40.53 (C-8), 38.67 (C-4), 38.54 (C-1), 38.17 (C-13), 37.00 (C-10), 36.91 (C-16), 34.21 (C-7), 30.43 (C-21), 29.52 (C-23), 29.27 (C-2), 27.19 (C-15), 25.54 (C-12), 22.79 (C-11), 20.70 (C-30), 18.17 (C-6), 18.03 (C-28), 15.32 (C-26), 14.69 (C-24), 13.67 (C-27) ppm (Figures S1–S6 [21,25]).

#### 3.4.2. Raucaffricine (**2**)

IR: ν_O-H_ at 3421.5 cm^−1^ (O-H) and ν_C = O_ at 1662.0 cm^−1^ (C=O). ^1^H-NMR (400 MHz, DMSO-d_6_): δ_H_ 7.55 (1H, d, *J* = 7.6 Hz, 9-H), 7.49 (1H, d, *J* = 7.2 Hz, 12-H), 7.39 (1H, dd, *J* = 7.6 Hz and *J* = 7.6 Hz, 10-H), 7.23 (1H, dd, *J* = 7.2 Hz and *J* = 7.6 Hz, 11-H), 5.65 (1H, q, *J* = 6.8 Hz, 19-H), 5.17 (1H, s, 21-H), 5.03 (1H, d, *J* = 4.4 Hz, 1′-H), 4.57 (1H, s, 17-H), 4.42 (2H, dd, *J* = 7.6 Hz and *J* = 8.8 Hz, 6′-H), 3.9-3.6 (1H, m, 5′-H), 3.18 (1H, dd, *J* = 5.6 Hz and *J* = 6 Hz, 5-H), 3.08 (1H, m, 15-H), 2.68 (1H, dd, *J* = 4.8 Hz and 4.4 Hz, 6-Hβ), 2.34 (1H, dd, *J* = 6 and *J* = 6.4 Hz, 16-H), 2.16 (3H, s, OCOCH_3_), 1.87 (1H, dd, *J* = 4 Hz and *J* = 12 Hz, 14-H_α_), 1.77 (1H, dd, *J* = 4.8 Hz and *J* = 4.8 Hz, 14-H_β_), 1.67 (3H, d, *J* = 11.6 Hz, 18-CH_3_), 1.45 (1H, d, *J* = 6.8 Hz, 6-H_α_) ppm. ^13^C-NMR (100 MHz, DMSO-d_6_): δ 184.42 (C-2), 170.13 (C-22), 156.86 (C-13), 137.94 (C-20), 137.09 (C-8), 127.06 (C-11), 125.81 (C-10), 124.29 (C-9), 122.76 (C-19), 120.85 (C-12), 99.32 (C-1′), 88.22 (C-21), 77.48 (C-3′,5′), 77.21 (C-17), 74.26 (C-2′), 70.58 (C-4′), 65.19 (C-7), 61.56 (C-6′), 55.24 (C-5), 50.45 (C-3), 48.49 (C-16), 37.32 (C-6), 27.47 (C-15), 24.67 (C-14), 21.32 (C-23), 13.28 (C-18) ppm. HRMS [M]^+^: *m*/*z* 513.2241; calcd. for C_27_H_32_N_2_O_8_: 513.2237 (Figures S7–S12 [21,28]).

#### 3.4.3. *N*-Methylsarpagine (**3**)

IR: 3312.90 (O-H), 2942.44 (C-H) and 2831.65 cm^−1^ (C-H). ^1^H-NMR (400 MHz, CD_3_OD): δ_H_ 7.11 (1H, d, *J* = 8.6 Hz, 12-H), 6.77 (1H, d, *J* = 2.0 Hz, 9-H), 6.68 (1H, dd, *J* = 8.9, 2.2 Hz, 11-H), 5.59 (1H, q, *J* = 6.8 Hz, 19-H), 4.41 (1H, d AB, *J* = 15.6 Hz, 21-H_α_), 4.16 (1H, d AB, *J* = 15.6 Hz, 21-H_β_), 3.48 (2H, d, *J* = 7.2 Hz, 17-H), 3.28 (3H, s, N-CH_3_), 3.15 (1H, dd, *J* = 12.4, 4.8 Hz, 6-H_β_), 3.0 (3H, s, N^+^-CH_3_), 2.95 (1H, dd, *J* = 2.0, 10.4 Hz, 15-H), 2.9 (1H, d, *J* = 15.2 Hz, 6-H_α_), 2.4 (1H, dd, *J* = 11.6, 10.8 Hz, 14-H_α_), 2.12-2.0 (2H, m, 16-H + 14-H_β_), 1.82 (s, OH), 1.63 (3H, d, *J* = 6.7 Hz, 18-H) ppm. ^13^C-NMR (100 MHz, CD_3_OD): δ_C_ 150.91 (C-10), 132.10 (C-20), 131.68 (C-13), 127.67 (C-8), 126.83 (C-2), 120.72 (C-19), 112.34 (C-12), 111.75 (C-11), 102.14 (C-9), 99.77 (C-7), 65.45 (C-5), 64.39 (C-17), 62.40 (C-21), 61.07 (C-3), 43.63 (C-16), 32.01 (C-14), 26.01 (C-15), 23.87 (C-6), 11.57 (C-18) ppm (Figures S13–S16 [21,27]).

#### 3.4.4. Spegatrine (**4**)

IR: 3352.1 cm^−1^ (O-H) and 1638.8 cm^−1^ (N-H). ^1^H-NMR (400 MHz, CD_3_OD): δH 7.24 (1H, d, *J* = 8.8 Hz, 12-H), 6.90 (1H, d, *J* = 2.0 Hz, 9-H), 6.78 (1H, dd, *J* = 2.2, 8.8 Hz, 11-H), 5.68 (1H, q, *J* = 6.8 Hz, 19-H), 4.45 (1H, d AB, *J* = 15.6 Hz, 21-H_α_), 4.23 (1H, d AB, *J* = 15.6 Hz, 21-H_β_), 3.58 (2H, d, *J* = 7.6 Hz, 17-H), 3.27 (1H, dd, *J* = 12.4, 4.8 Hz, 6-H_β_), 3.15 (3H, s, N^+^-CH_3_), 3.12 (1H, dd, *J* = 2.0, 10.4 Hz, 15-H), 3.04 (1H, d, *J* = 17.2 Hz, 6-H_α_), 2.54 (1H, dd, *J* = 11.6, 10.8 Hz, 14-H_α_), 2.23-2.13 (2H, m, 16-H + 14-H_β_), 1.97 (s, OH), 1.74 (3H, d, *J* = 6.8 Hz, 18-H) ppm. ^13^C-NMR (100 MHz, CD_3_OD): δ_C_ 150.93 (C-10), 132.10 (C-20), 131.66 (C-13), 127.68 (C-8), 126.83 (C-2), 120.71 (C-19), 112.35 (C-12), 111.73 (C-11), 102.13 (C-7), 99.77 (C-9), 65.46 (C-5), 64.38 (C-21), 62.40 (C-17), 61.08 (C-3), 46.67 (N^+^-CH3), 43.62 (C-16), 32.00 (C-14), 26.01 (C-15), 23.87 (C-6), 11.56 (C-18) ppm. HRMS [M]^+^: *m*/*z* 325.1912; calcd. for C_20_H_25_N_2_O_2_^+^: 325.1911 (Figures S17–S21 [21,27]).

### 3.5. Antioxidant Activities

The crude extract, fractions and pure compounds were evaluated for antioxidant activity. Initially, the crude extract and fractions were tested. Then the compounds isolated from the fractions were tested. The highest IC_50_ or IC_0.5_ values indicate the lowest antioxidant activity while the lowest IC_50_ or IC_0.5_ values imply the highest antioxidant activity.

#### 3.5.1. Free Radical Scavenging Assay (DPPH)

The DPPH free radical scavenging ability of the crude extract and fractions F_1_, F_2_, F_3_, F_4_ and F_5_ was determined according to the spectrophotometric method of Anokwuru et al. [29]. A 125 mM DPPH/methanol solution was prepared by dissolving 10 mg DPPH in 200 mL methanol. The 96-well plates were filled with 100 µL distilled water per well. One hundred µL of the crude extract, fractions F_1_, F_2_, F_3_, F_4_ and F_5_, compound **4** and 100 µL methanol were added in triplicate into the first three wells followed by serial dilution. Two hundred μL 0.3 M DPPH/methanol was added to each well containing the mixtures. The 96-well plate was kept in the dark for 30 min and the absorbance was measured using a VersaMax^TM^ tuneable microplate reader at 517 nm.

The percentage radical scavenging was calculated by the following formula:

% Free RSA = [(ADPPH − Asample)/(ADPPH)] × 100(1)

#### 3.5.2. Reducing Power

The reducing power was determined according to the method of Anokwuru et al. [29]. A 0.2 M (pH 6.6) sodium phosphate buffer (50 µL) and 50 µL of the crude extract, fractions F_1_, F_2_, F_3_, F_4_ and F_5_, and compound **4** were added in triplicate in the first three wells of a 96-well plate, followed by serial dilution. Fifty µL of a 1% aqueous potassium hexacyanoferrate(III) [K_3_Fe(CN)_6_] solution was added to each well. The 96-well plates were placed in an incubator for 20 min at 50 °C. After incubation, 50 µL of 10% trichloroacetic acid solution was added to each well. Eighty µL of each mixture was transferred to another 96-well plate and 80 µL of distilled water, followed by 16 µL ferric chloride (0.1% *w*/*v*) was added. Absorbance was measured using a VersaMax™ tuneable microplate reader at 700 nm.

### 3.6. Antitrypanosomal Activity

To assess trypanocidal activity, compounds were added to in vitro cultures of *T. b. brucei* (strain Lister 427) in 96-well plates (triplicate; 2.4 × 10^4^ parasites/well) at a fixed concentration of 20 µM for pure compounds (**2** and **4**) or 25 µg/mL for the crude extract and fractions F_1_, F_2_, F_3_, F_4_ and F_5_. The culture medium consisted of Iscove’s Modified Dulbecco’s Medium containing 25 mM HEPES (4-(2-hydroxyethyl)-1-piperazineethanesulfonic acid) (IMDM; Thermo Fisher Scientific) supplemented with 10% fetal bovine serum (Biowest), penicillin/streptomycin (Lonza) and HMI-9 supplement (0.05 mM bathocuproine disulfonic acid, 1.5 mM cysteine, 1.25 mM pyruvic acid, 0.09 mM uracil, 0.09 mM cytosine, 0.16 mM thymidine, 0.014% 2-mercaptoethanol, 1 mM hypoxanthine; all reagents from Sigma-Aldrich) [30]. After an incubation period of 24 h at 37 °C in a 5% CO_2_ humidified incubator, resazurin (Sigma-Aldrich) was added to a final concentration of 0.05 mM and incubation continued for a further 24 h, after which conversion of resazurin to resorufin was measured in a Spectramax M3 fluorescence plate reader (Molecular Devices; Exc_560_/Em_590_). The % cell viability in wells containing test samples was calculated relative to untreated control wells using the fluorescence readings, after subtracting background fluorescence in wells without cells. For dose-response experiments, parasites were cultured in the presence of 3-fold serial dilutions of the test samples and % viability plotted against log (compound concentration) to obtain IC_50_ values by non-linear regression (GraphPad Prism v.5.02). Pentamidine (an existing drug for treatment of trypanosomiasis) was used as the positive control.

### 3.7. Anti-Proliferation Activity

To assess cytotoxicity, the samples were incubated at a fixed concentration of 20 μM for pure compounds (**2** and **4**) and 50 μg/mL for crude extract and fractions F_1_, F_2_, F_3_, F_4_ and F_5_ in 96-well plates seeded 24 h earlier with HeLa cells (Cellonex; 2 × 10^4^ cells per well). Incubation was carried out for 48 h in a 37 °C 5% CO_2_ humidified incubator. The culture medium consisted of Dulbecco′s modified Eagle′s medium (DMEM; Thermo Fisher Scientific) supplemented with 10% fetal bovine serum (Biowest) and penicillin/streptomycin/amphotericin (Lonza). Resazurin (Sigma-Aldrich) was added to a final concentration of 0.05 mM and incubation continued for 2 h, after which fluorescence (Exc_560_/Em_590_) was measured in a Spectramax M3 plate reader (Molecular Devices) (San Jose, CA, USA) [31]. Fluorescence values were converted to % cell viability relative to untreated control wells, after subtracting background readings obtained from wells without cells.

### 3.8. Statistical Analysis

The collected data were entered on an Excel spreadsheet and statistical analysis was undertaken using the SPSS package (Chicago, IL, USA). The absorbance and reduced concentrations were used to calculate the linear regression. One-way analysis of variance (ANOVA) was used to compare mean values of the crude extract, fractions F_1_, F_2_, F_3_, F_4_ and F_5_, and spegatrine (**4**) obtained in the antioxidant tests; *p* < 0.05 was considered statistically significant. For dose-response experiments, parasites were cultured in the presence of 3-fold serial dilutions of the test samples and % viability plotted against log (compound concentration) to obtain IC_50_ values by non-linear regression (GraphPad Prism v.5.02). Pentamidine (an existing drug for treatment of trypanosomiasis) was used as the positive control.

## 4. Conclusions

The main objective of this study was to determine the in vitro antioxidant, antitrypanosomal and anti-proliferation activities of *R. caffra.* The proven antioxidant and antiparasitic activities of the plant extract and its fractions, and their non-cytotoxicity, support the traditional medicinal use of *R. caffra* and also shows that traditional medicine is a reliable source of knowledge for the development of new drugs. The study also shows that the major constituent raucaffricine (**2**) does not contribute to the biological activities evaluated. Further research should be carried out to isolate, identify, characterize and elucidate the structures of more of the bioactive compounds present so as to have a complete picture in terms of its in vitro antioxidant and antitrypanosomal activities.

## Figures and Tables

**Table 1:** Antioxidant activity of crude extract, fractions and pure compound.

Sample	DPPH IC_50_ (mg/mL)	Reducing Power IC_0.5_ (mg/mL)
Crude extract	0.213 ± 0.068 ^a^	1.226 ± 0.443 ^a^
F_1_	0.653 ± 0.307 ^a,b,c^	2.036 ± 0.266 ^b,c^
F_2_	0.413 ± 0.195 ^a^	1.282 ± 0.036 ^a^
F_3_	1.143 ± 0.478 ^b,c^	2.151 ± 0.372 ^b,c^
F_4_	0.022 ± 0.003 ^a,d,e^	0.518 ± 0.044 ^e^
F_5_	0.036 ± 0.007 ^a,d,e^	1.076 ± 0.136 ^a,e^
Spegatrine (**4**)	0.119 ± 0.067 ^a^	0.715 ± 0 ^a,e^
Gallic acid	0.045 ± 0.018 ^a^	0.115 ± 0.007 ^e^

Notes: A different superscript letter indicates significant difference using one-way ANOVA at *p* < 0.05. Data (*n* = 3) expressed as mean ± standard deviation. For DPPH (2,2-diphenyl-1-picrylhydrazyl) free radical scavenging activity: For DPPH (2,2-diphenyl-1-picrylhydrazyl) free radical scavenging: ^a^—Crude extract was only significantly different from Fraction F_3_; ^a,b,c^—Fraction F_1_ was significantly different from Fraction F_4_ and Fraction F_5_, and ^a,d,e^—Fraction F_4_ was not significantly different from Fraction F_5_. For Reducing power activity: ^b,c^—Fraction F_1_ was not significantly different from Fraction F_3_ and ^a,e^—Fraction F_5_ was not significantly different from spegatrine (**4**).

## Supplementary Materials

Supplementary materials are available online.
