# Supplementary material for: In Vitro Studies on Antioxidant and Anti-Parasitic Activities of Compounds Isolated from Rauvolfia caffra Sond
Source: Molecules. 2020 Aug 20;25(17):3781. doi: 10.3390/molecules25173781 (PMC7503976; doi:10.3390/molecules25173781)
Supplement: Supplementary file 1 [file molecules-25-03781-s001.docx]

Supplementary Materials

In Vitro Studies on Antioxidant and Anti-Parasitic Activities of Compounds Isolated from *Rauvolfia caffra* Sond

Dorcas B. Tlhapi ^1^, Isaiah D. I. Ramaite ^1,^*, Chinedu P. Anokwuru ^1^, Teunis van Ree ^1^ and Heinrich C. Hoppe ^2^

^1^ Department of Chemistry, University of Venda, Private Bag X5050, Thohoyandou 0950, South Africa; dorcastlhapi@gmail.com (D.B.T.); anokwuruchi@gmail.com (C.P.A.); Teuns.VanRee@univen.ac.za (T.v.R.)

^2^ Department of Biochemistry and Microbiology, Rhodes University, Grahamstown 6140, South Africa; H.Hoppe@ru.ac.za

***** Correspondence: Isaiah.Ramaite@univen.ac.za; Tel.: +27-(0)1-5962-8262

**
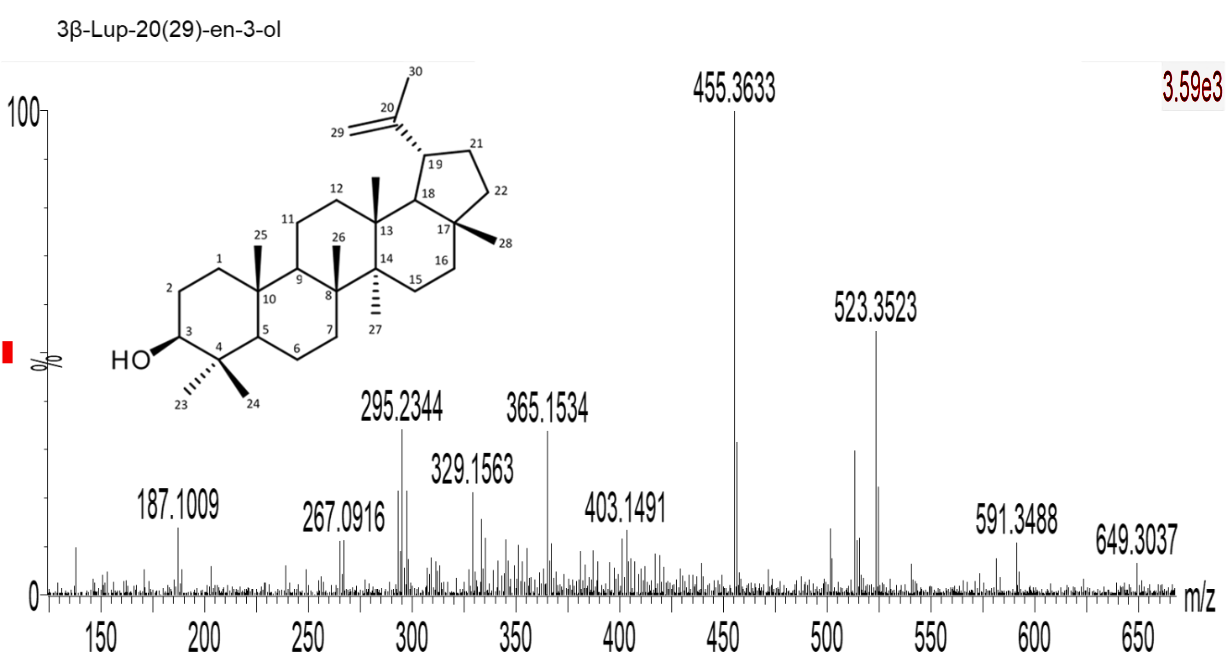
**

**Figure S1.** Mass spectrum of lupeol (**1**) [21,25].

**
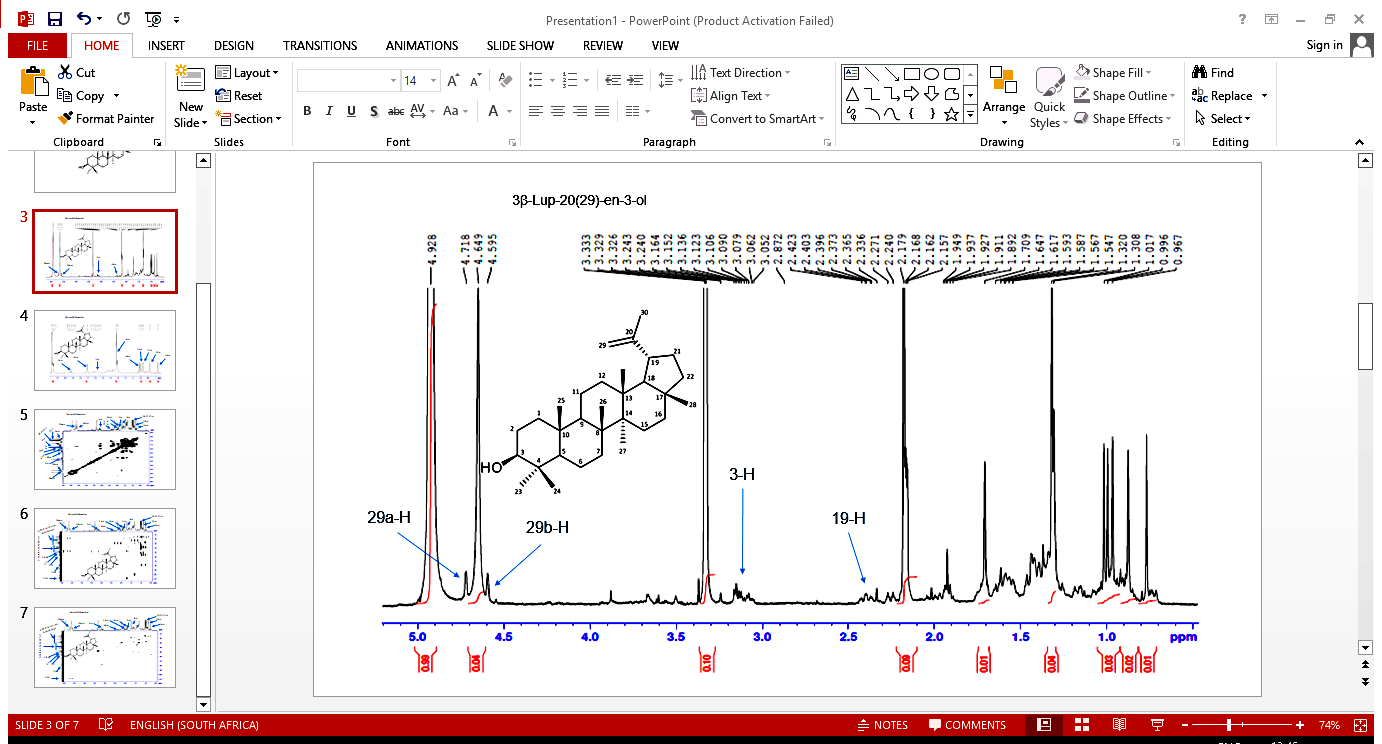
**

**Figure S2.** Expanded ^1^H-NMR spectrum of lupeol (**1**) [21,25].

**
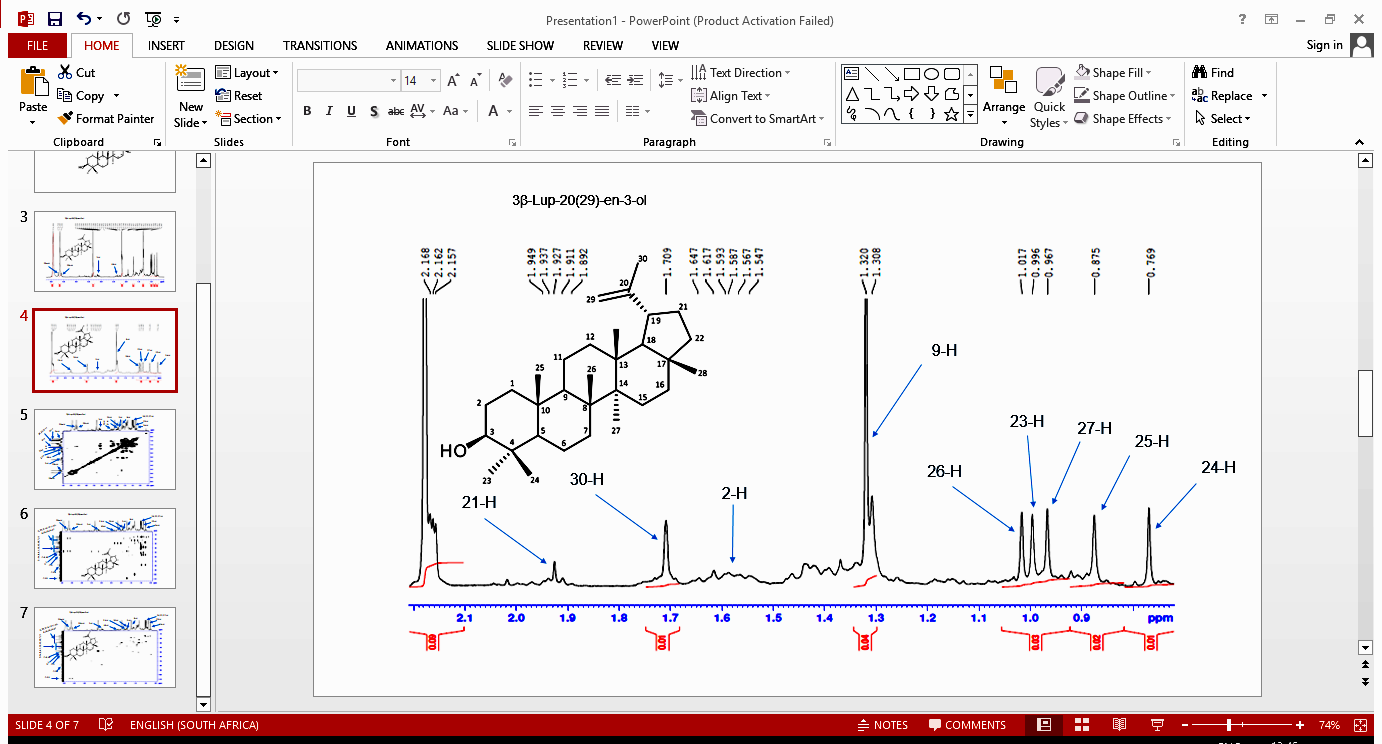
**

**Figure S3.** Expanded ^1^H-NMR spectrum of lupeol (**1**) [21,25].

**
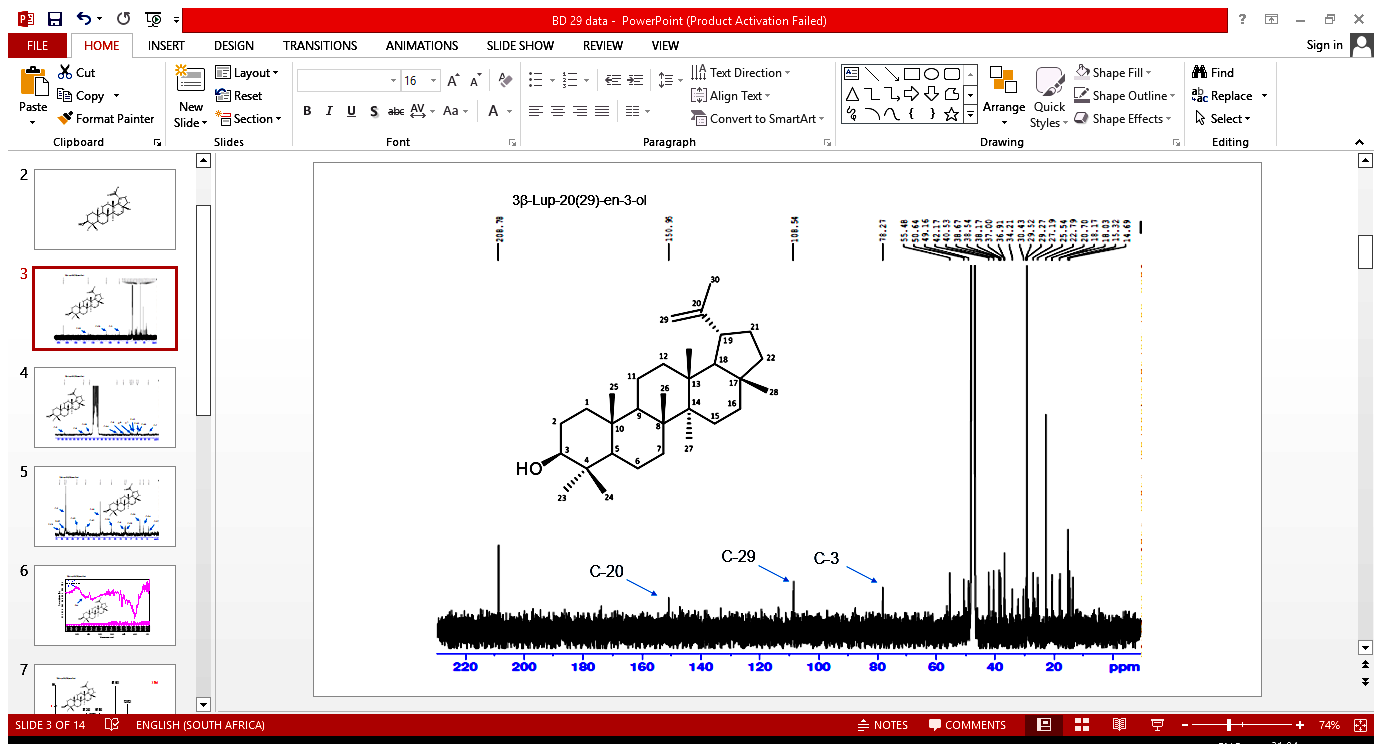
**

**Figure 4.** ^13^C-NMR spectrum of lupeol (**1**) [21,25].


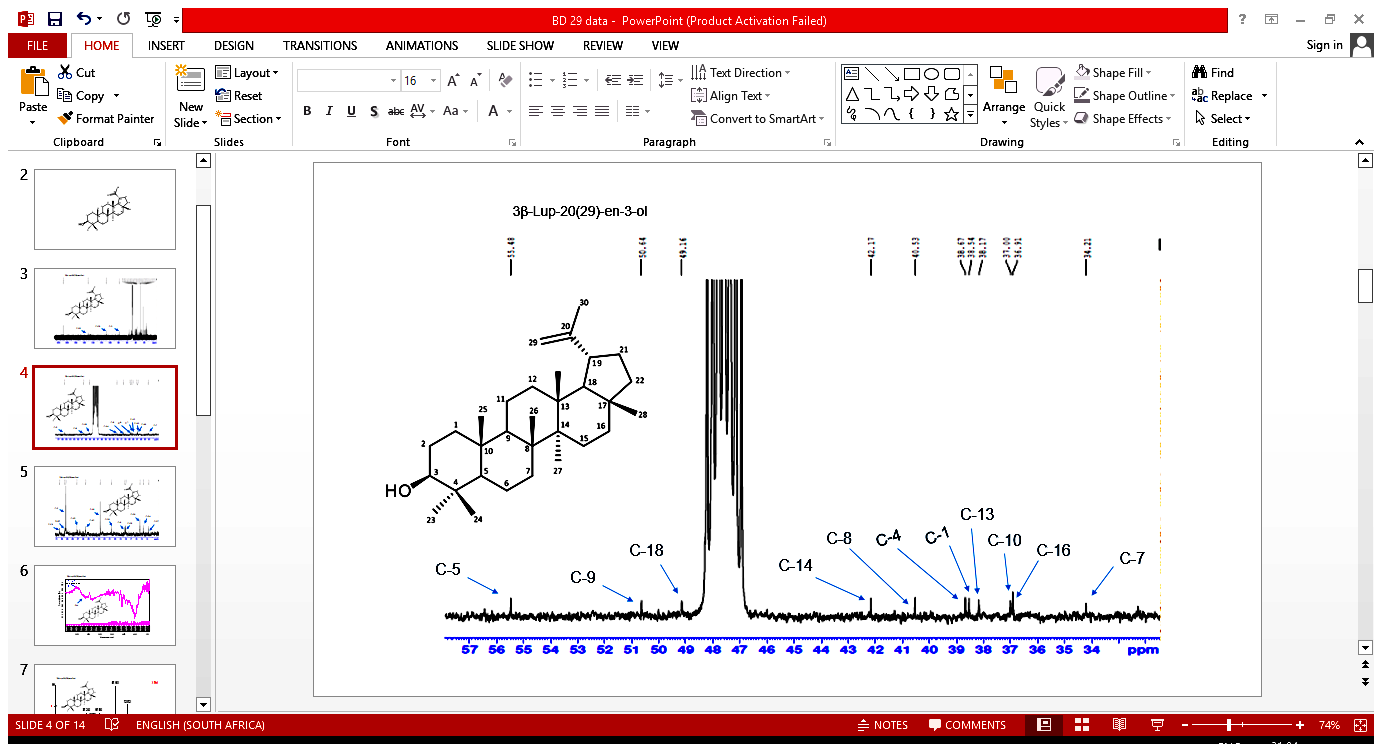


**Figure 5.** ^13^C-NMR spectrum of lupeol (**1**) [21,25].


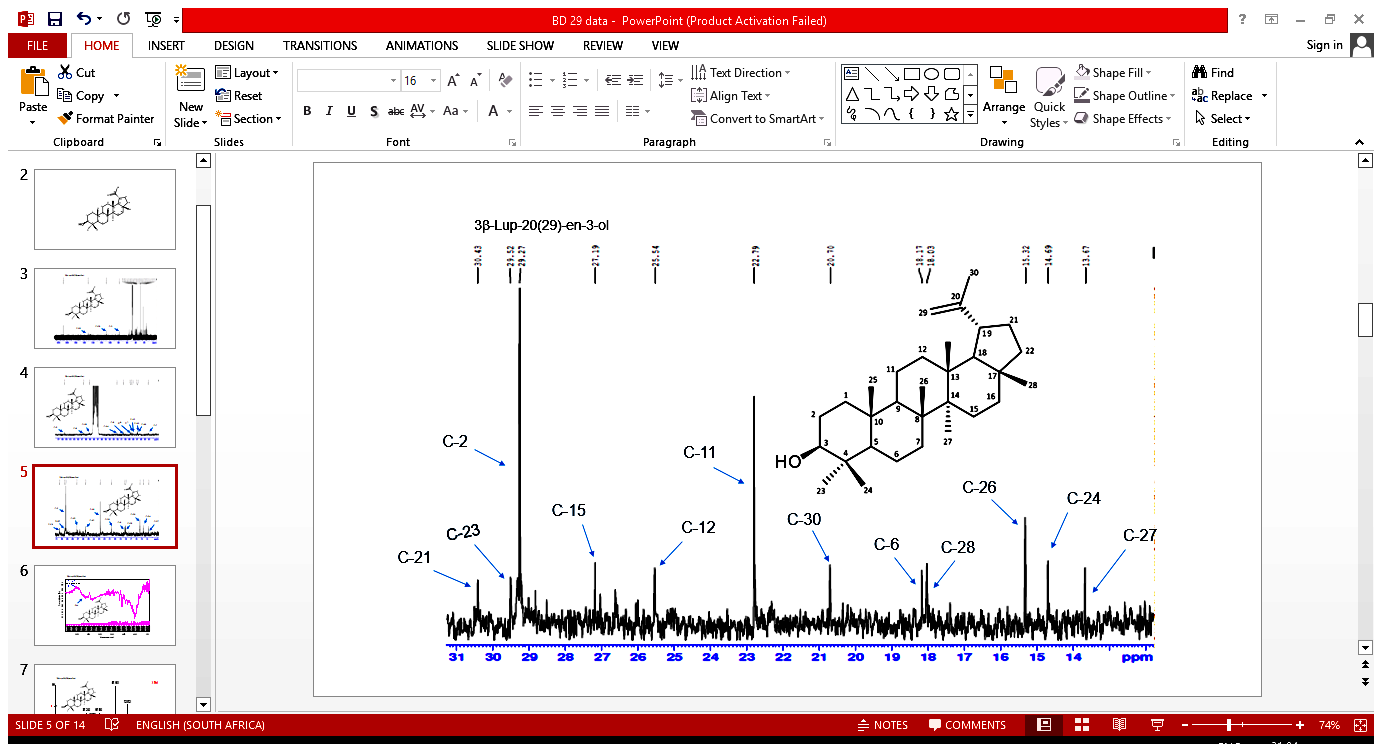


**Figure 6.** ^13^C-NMR spectrum of lupeol (**1**) [21,25].


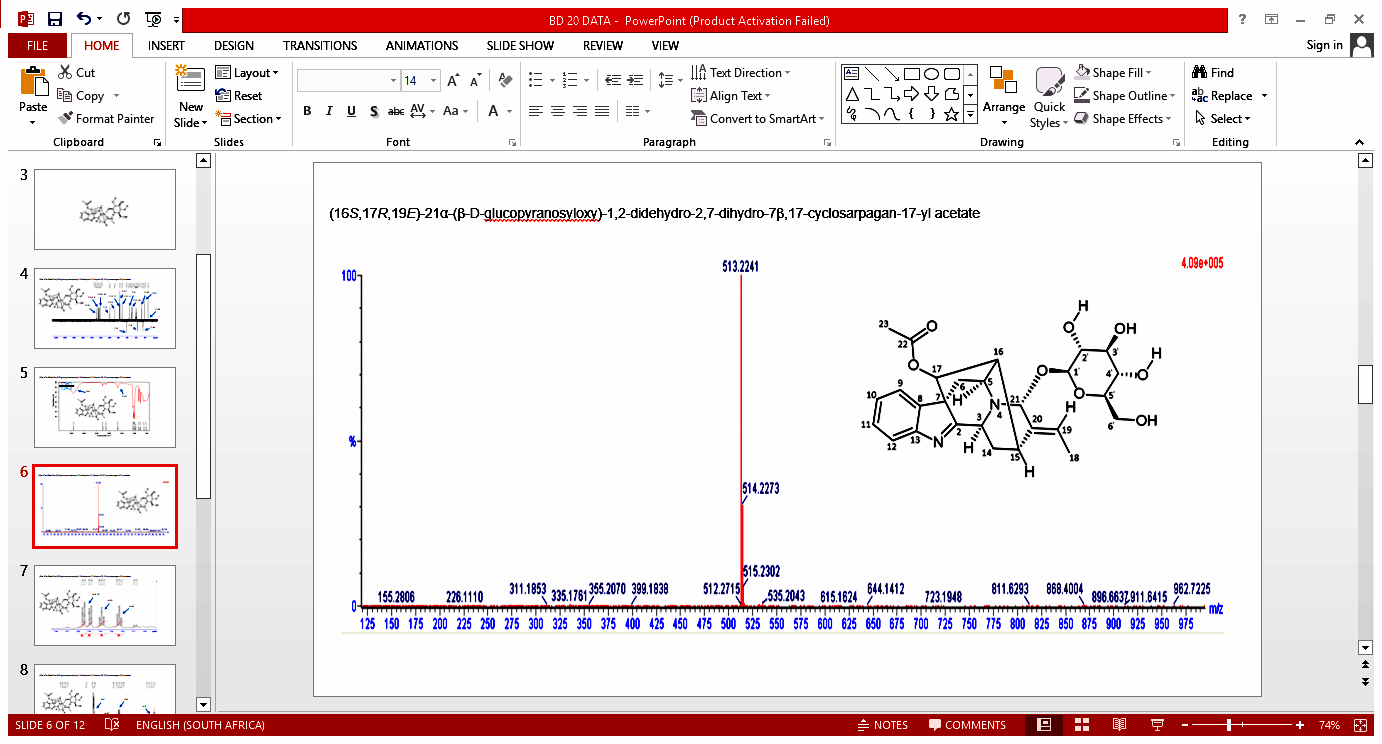


**Figure 7.** Mass spectrum of raucaffricine (**2**) [21,28].


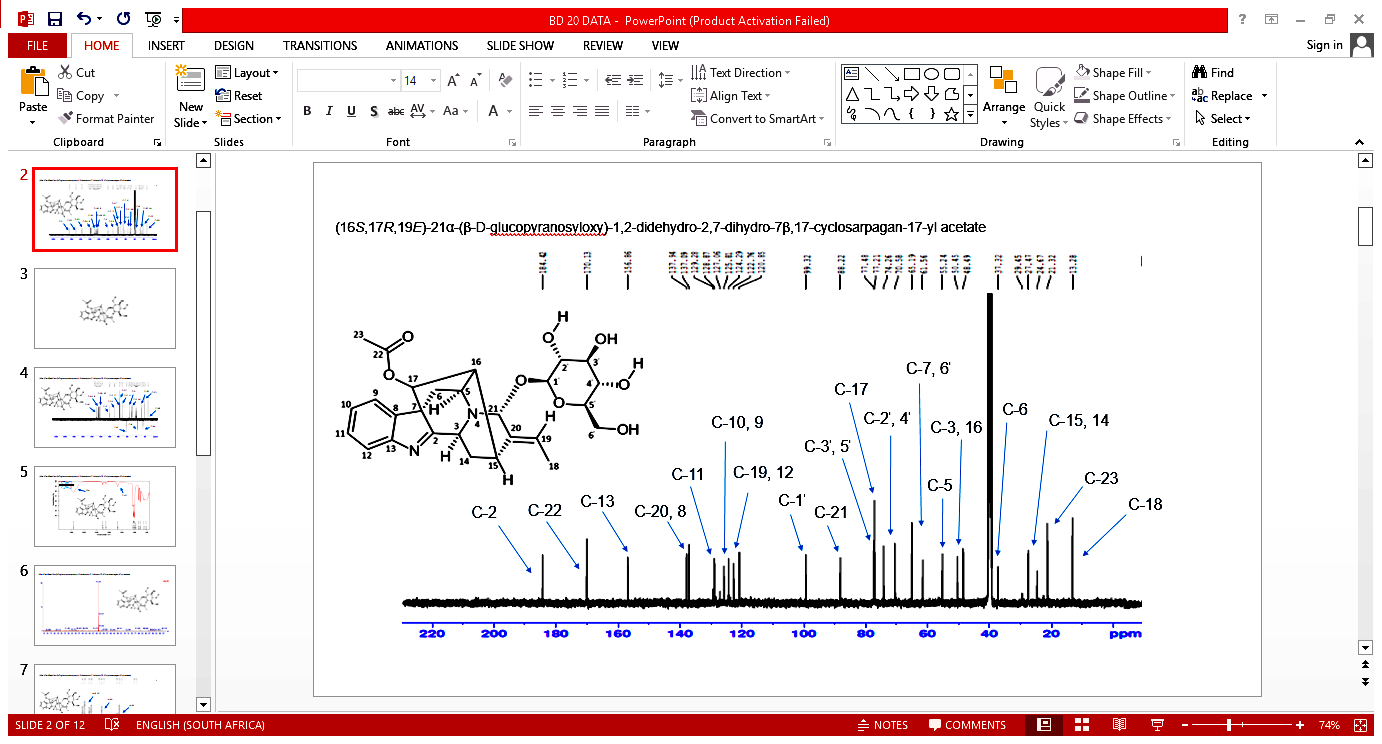


**Figure 8.** ^13^C-NMR spectrum of raucaffricine (**2**) [21,28].


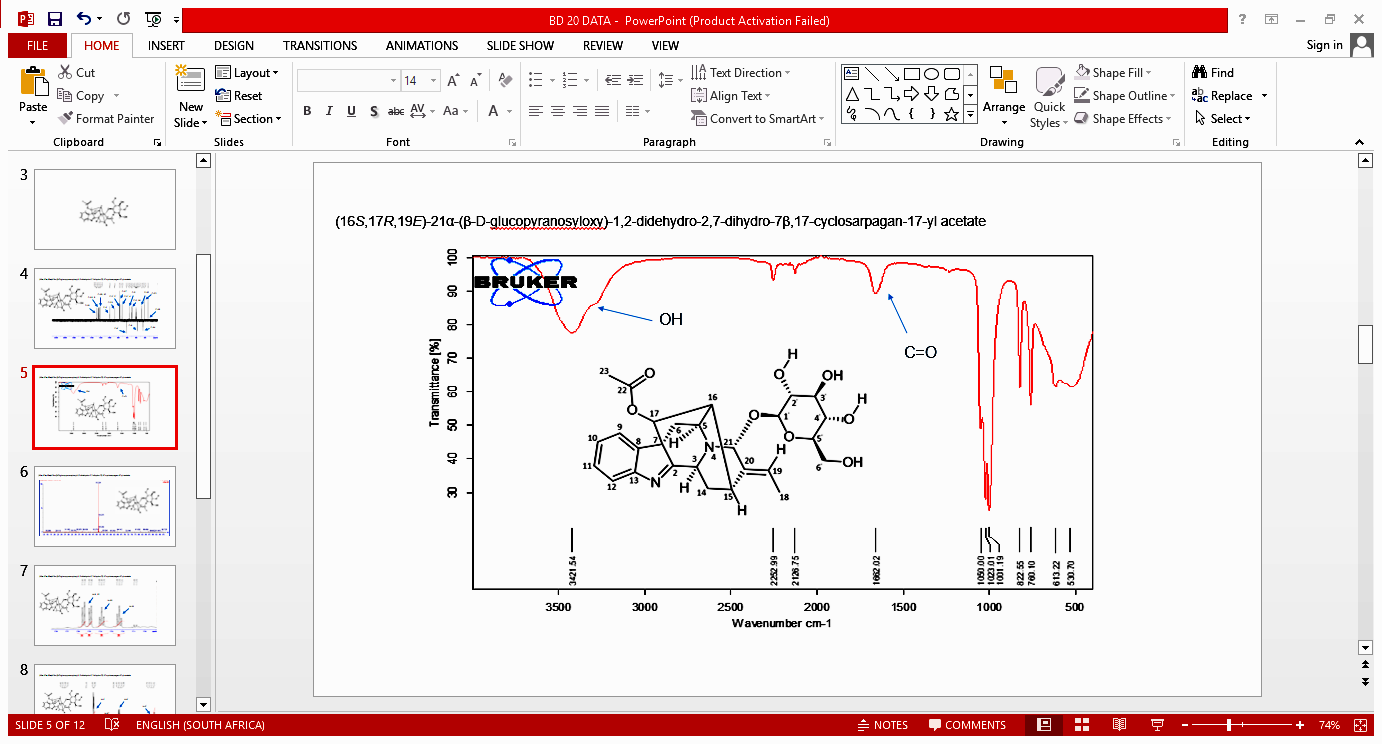


**Figure 9.** IR spectrum of raucaffricine (**2**) [21,28].


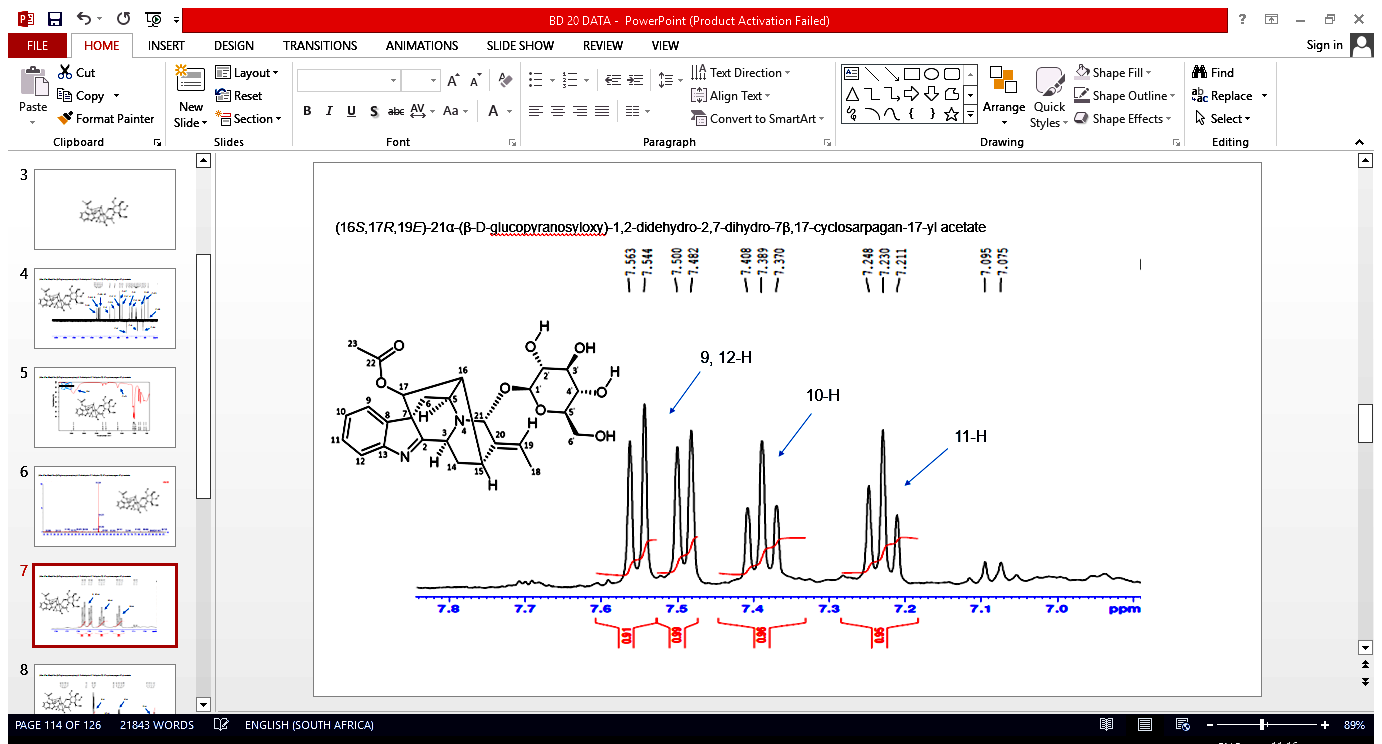


**Figure 10.** Expanded ^1^H-NMR spectrum of raucaffricine (**2**) [21,28].


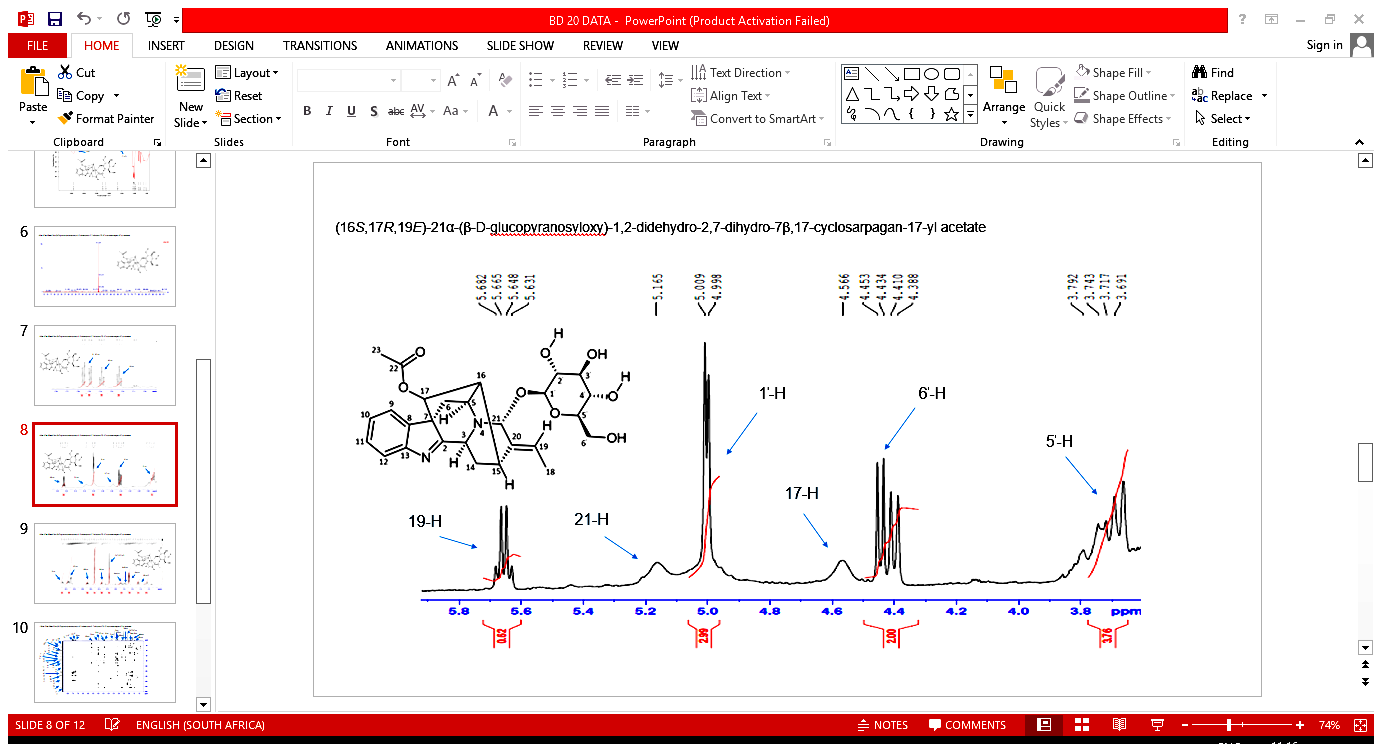


**Figure 11.** Expanded ^1^H-NMR spectrum of raucaffricine (**2**) [21,28].


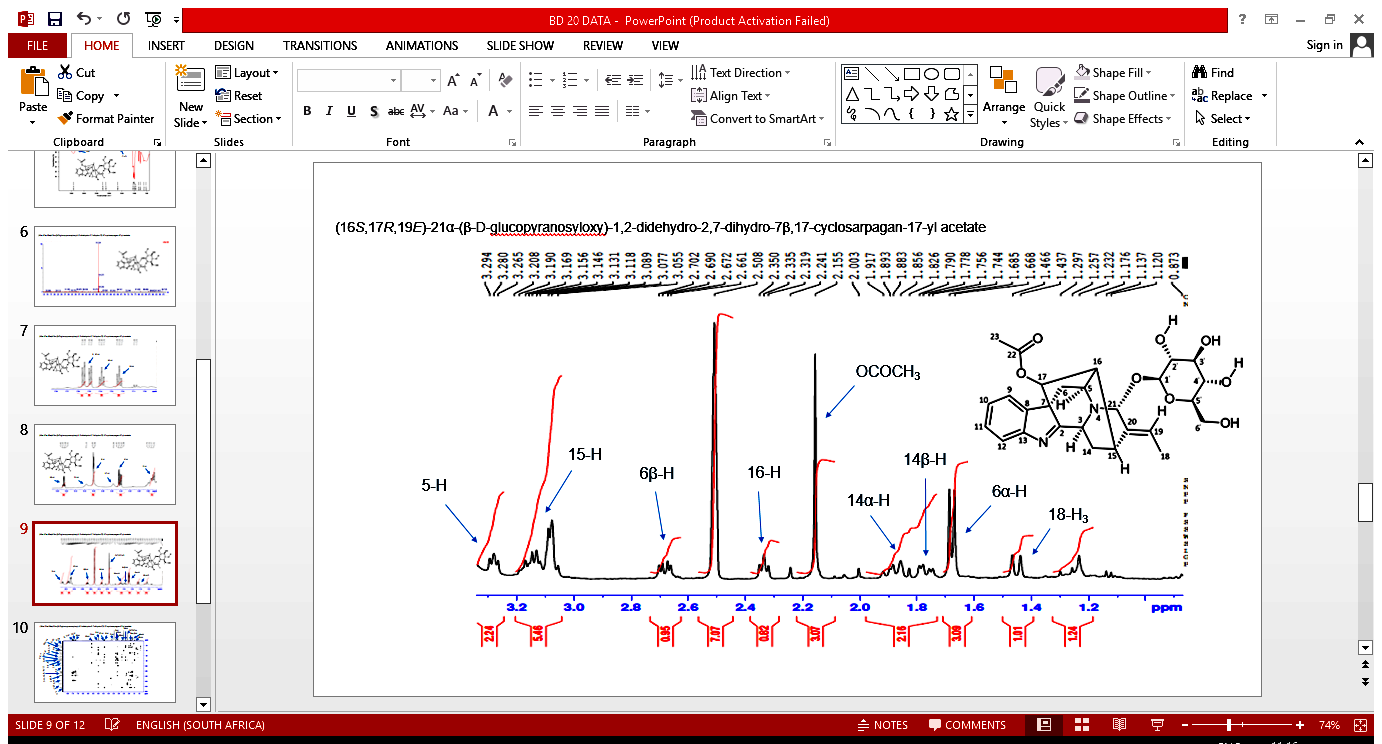


**Figure 12.** Expanded ^1^H-NMR spectrum of raucaffricine (**2**) [21,28].


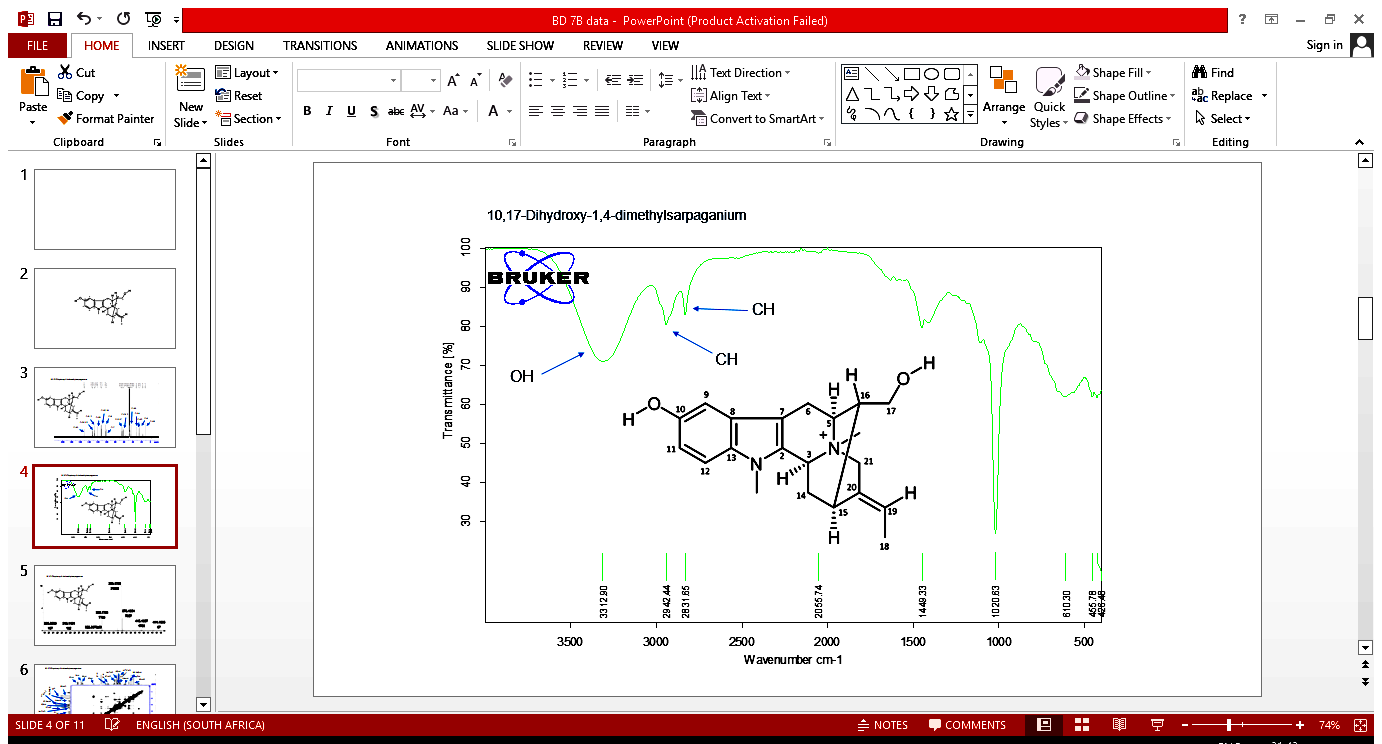


**Figure 13.** IR spectrum of *N*-methylsarpagine (**3**) [21,27].


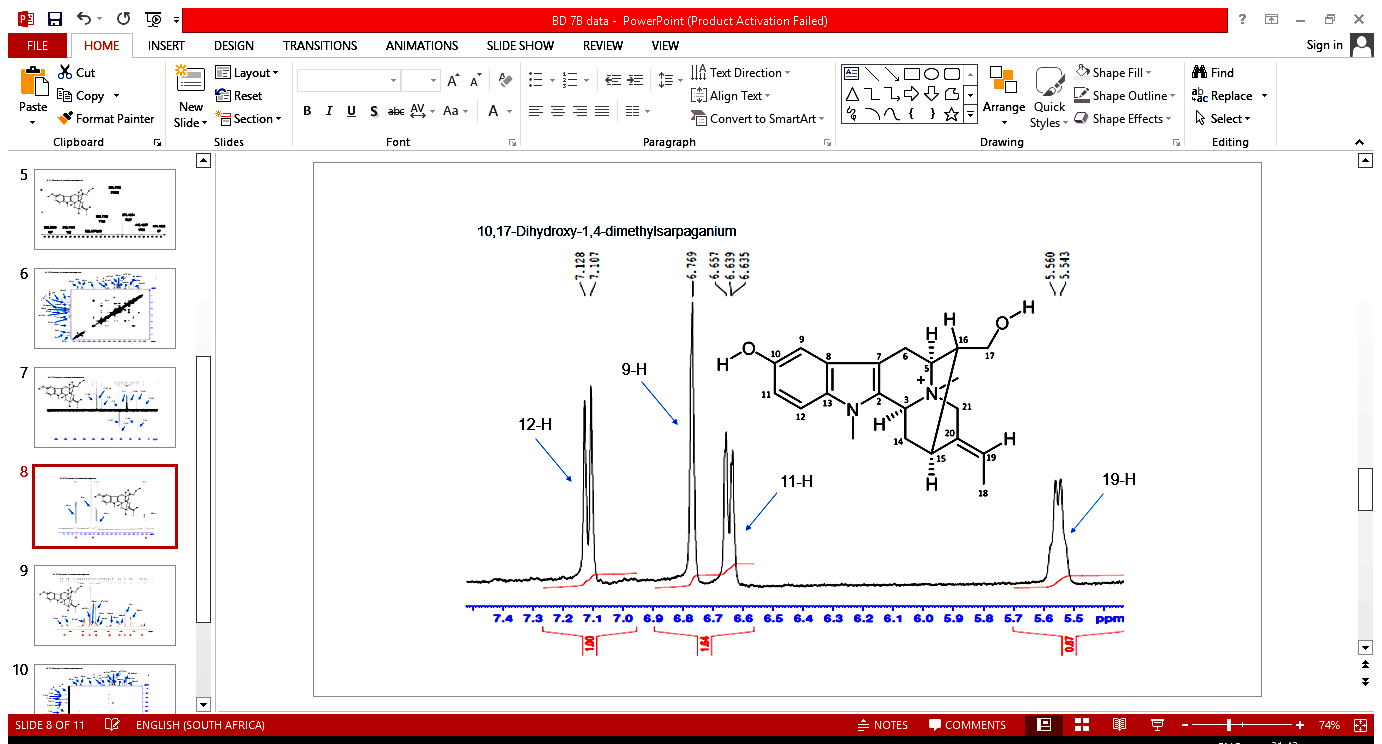


**Figure 14.** Expanded ^1^H-NMR spectrum of *N*-methylsarpagine (**3**) [21,27].


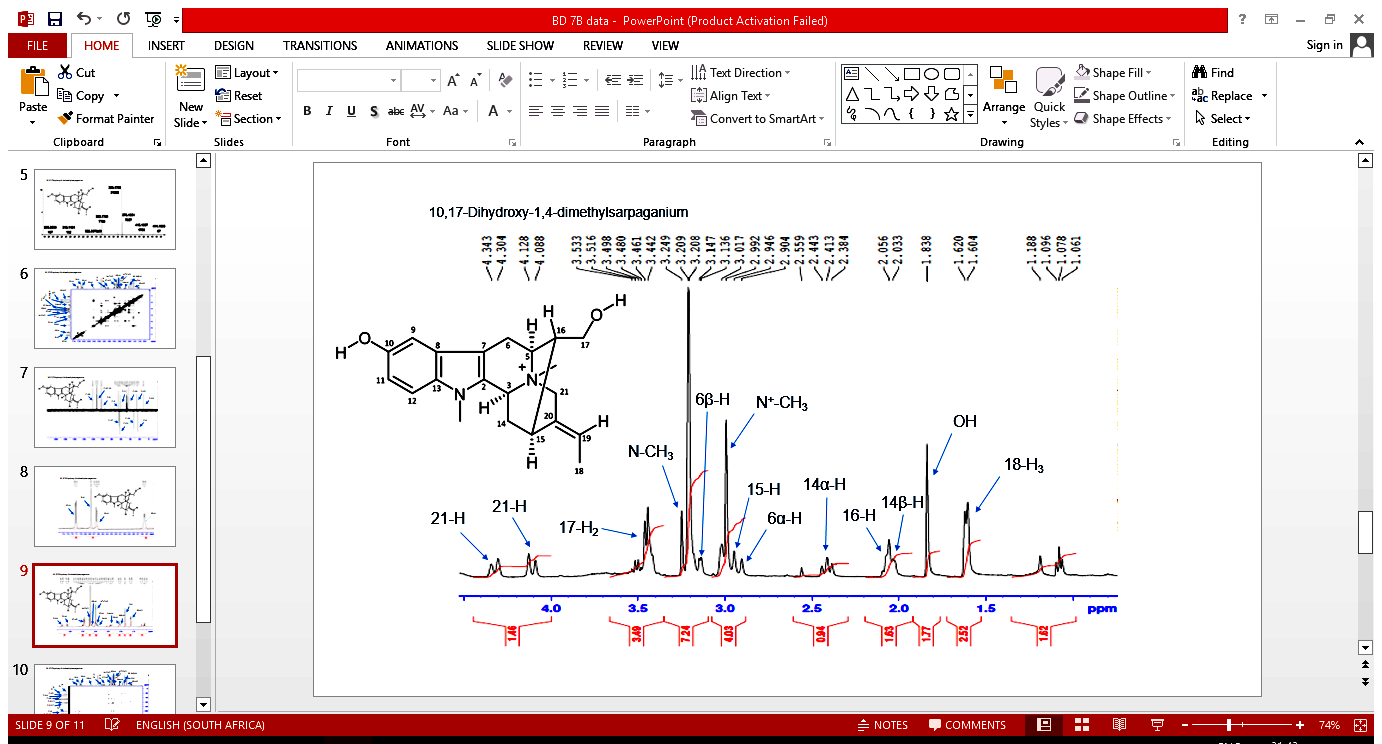


**Figure 15.** Expanded ^1^H-NMR spectrum of *N*-methylsarpagine (**3**) [21,27].

**
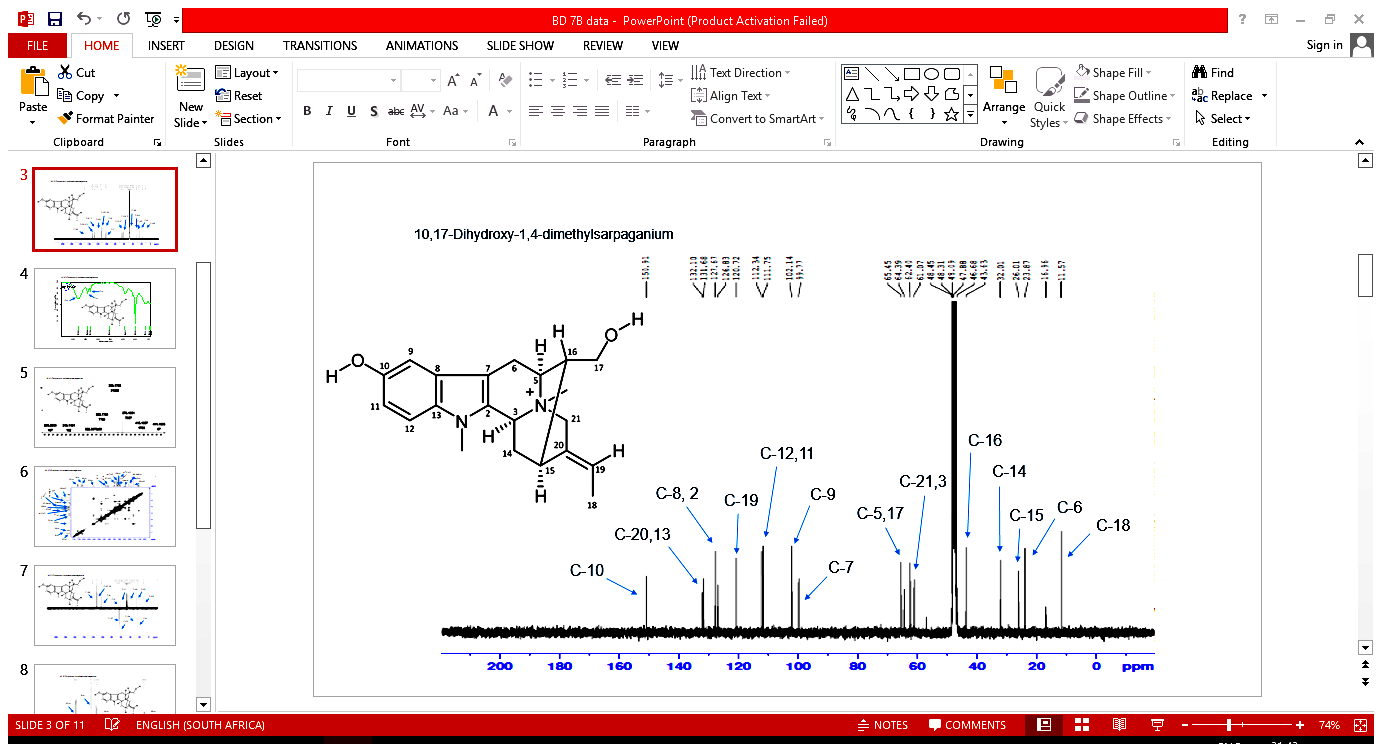
**

**Figure S16.** ^13^C-NMR spectrum of *N*-methylsarpagine (**3**) [21,27].


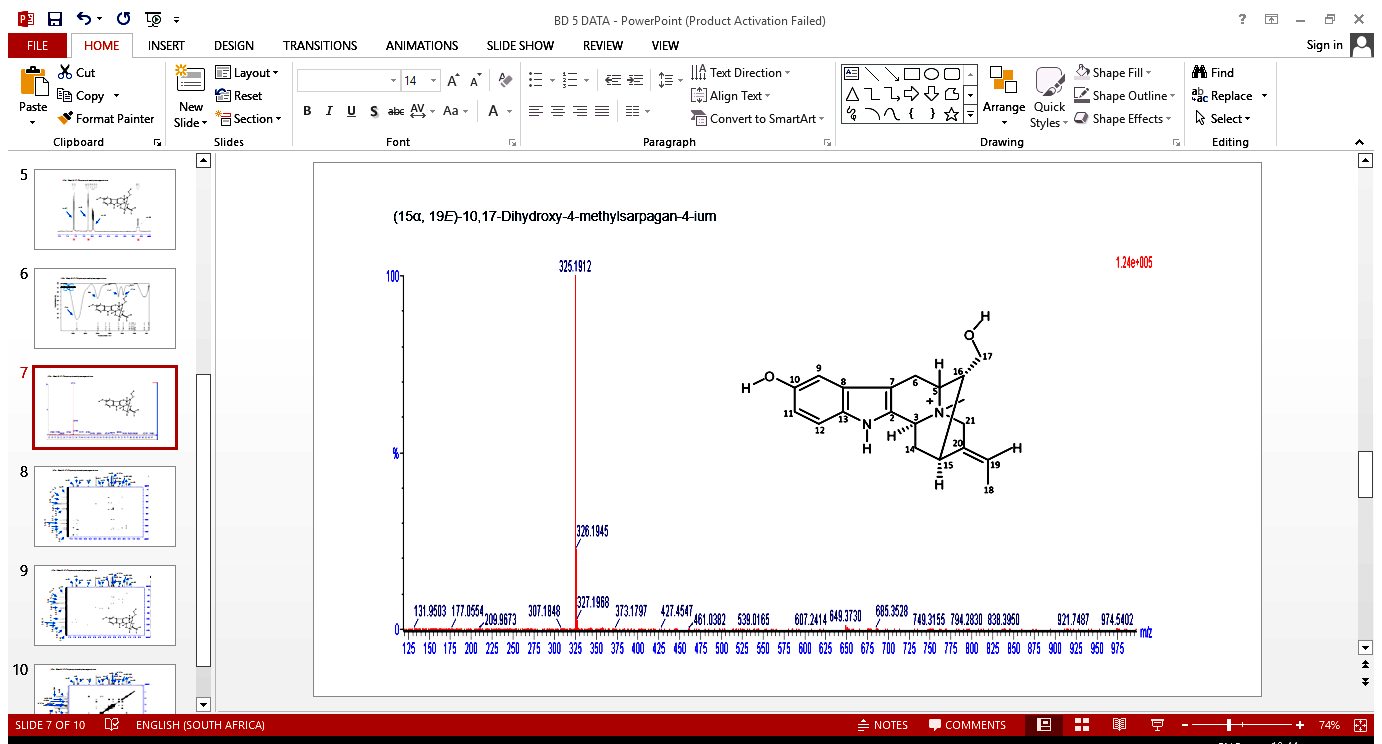


**Figure 17.** Mass spectrum of spegatrine (**4**) [21,27].


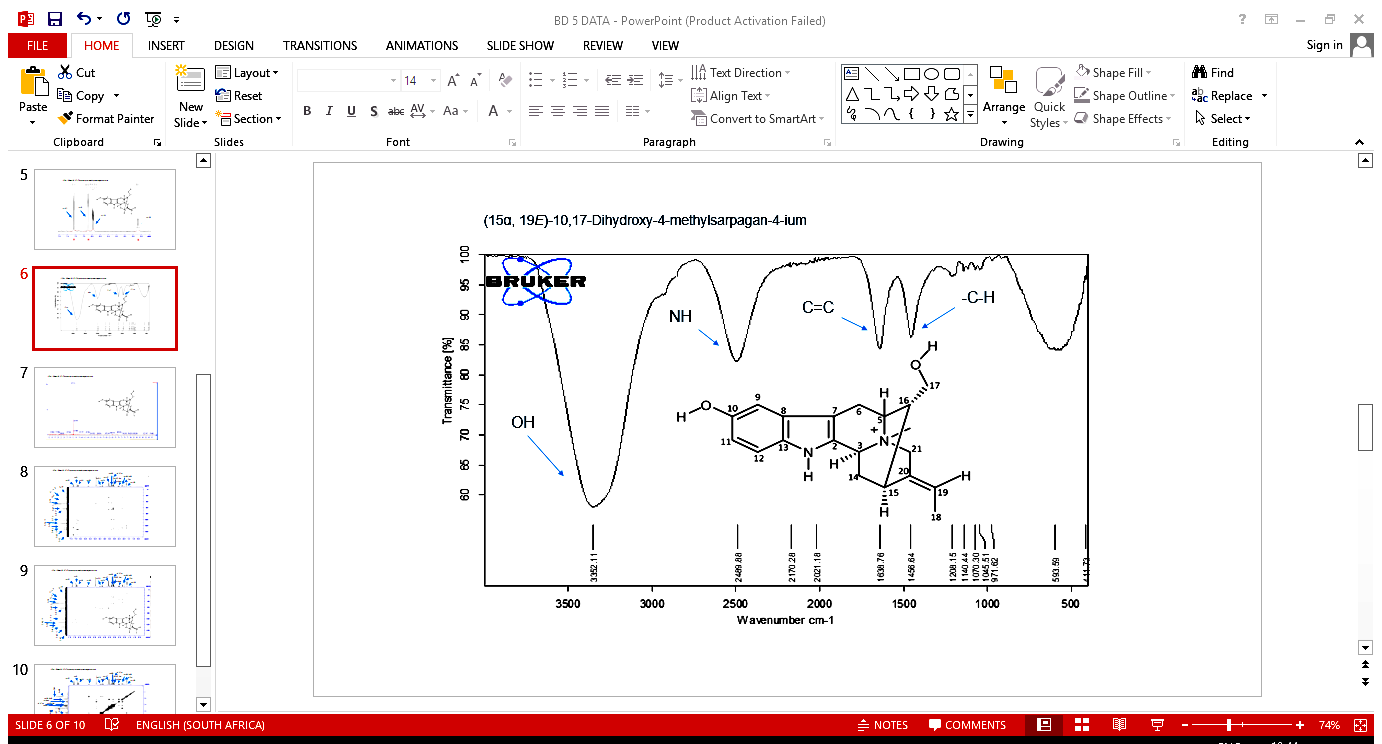


**Figure 18.** IR spectrum of spegatrine (**4**) [21,27].

**
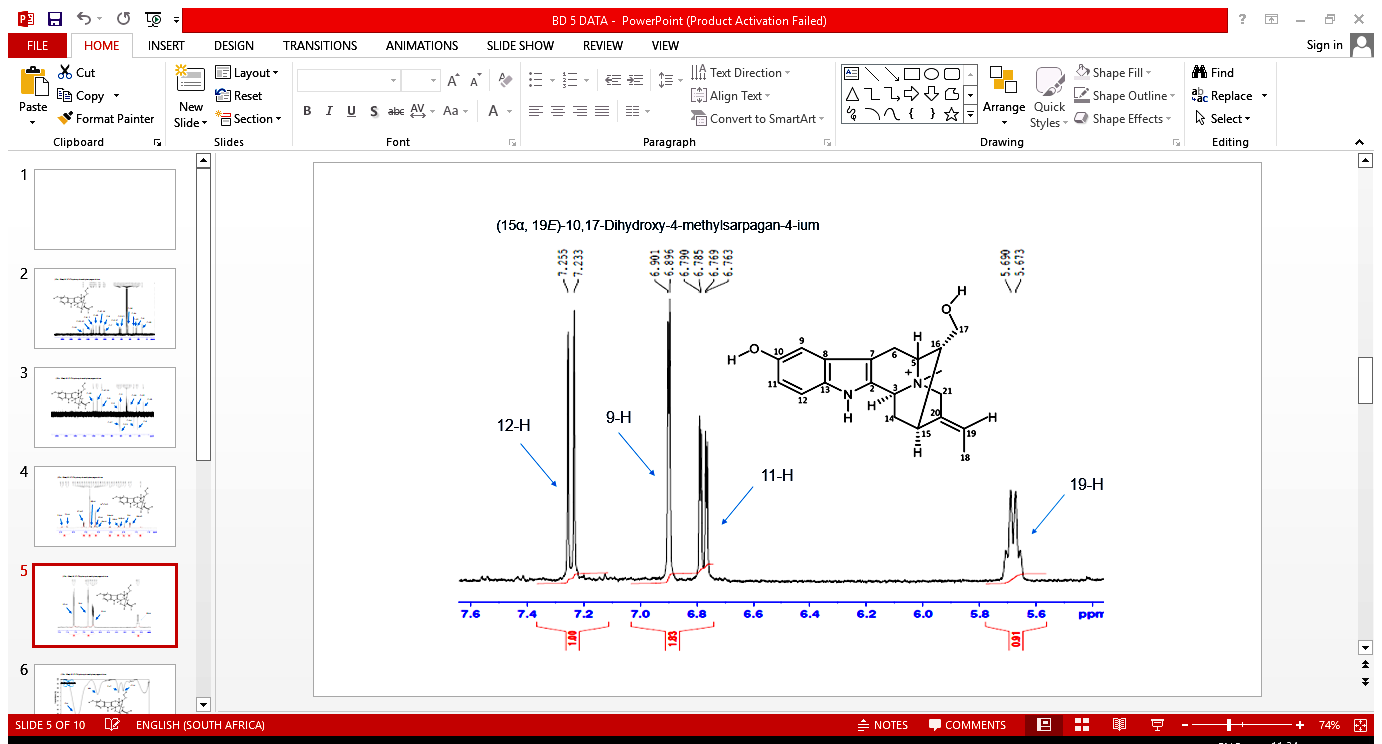
**

**Figure S19.** Expanded ^1^H-NMR spectrum of spegatrine (**4**) [21,27].

**
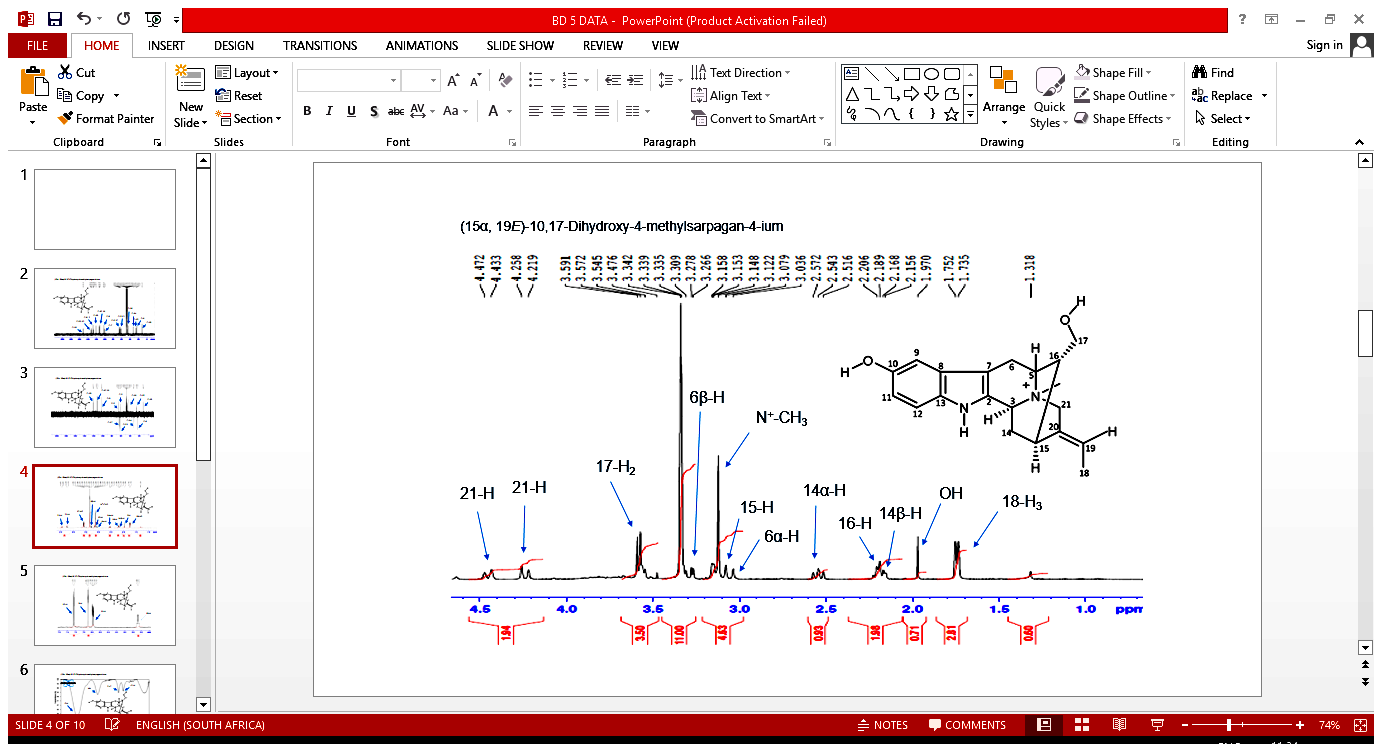
**

**Figure S20.** Expanded ^1^H-NMR spectrum of spegatrine (**4**) [21,27].

**
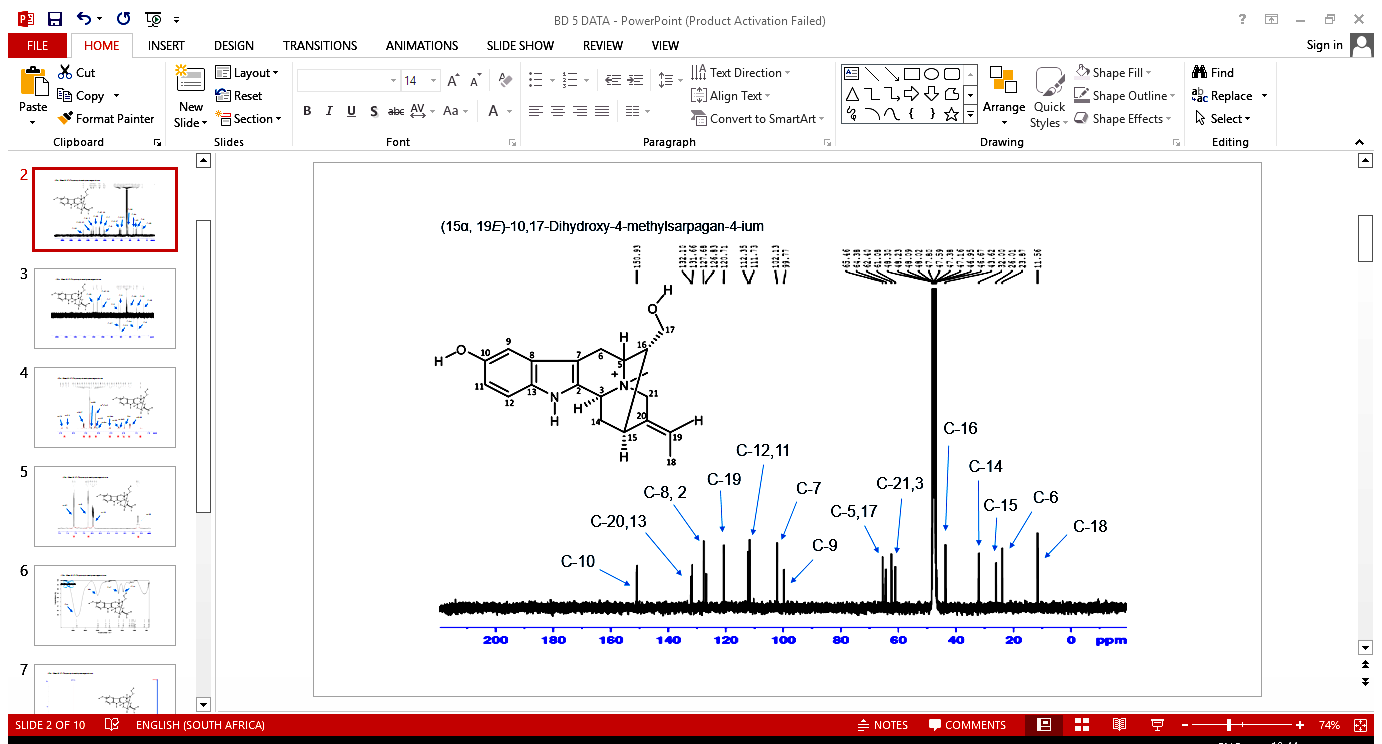
**

**Figure S21.** ^13^C-NMR spectrum of spegatrine (**4**) [21,27].
